# Preosteocytes/Osteocytes Have the Potential to Dedifferentiate Becoming a Source of Osteoblasts

**DOI:** 10.1371/journal.pone.0075204

**Published:** 2013-09-06

**Authors:** Elena Torreggiani, Brya G. Matthews, Slavica Pejda, Igor Matic, Mark C. Horowitz, Danka Grcevic, Ivo Kalajzic

**Affiliations:** 1 Department of Reconstructive Sciences, University of Connecticut Health Center, Farmington, Connecticut, United States of America; 2 Department of Orthopaedics and Rehabilitation, Yale University School of Medicine, New Haven, Connecticut, United States of America; 3 Department of Physiology and Immunology, University School of Medicine, Zagreb, Croatia; The University of Adelaide, Australia

## Abstract

Presently there is no clear evidence for the ability of mature osteogenic lineage cells to dedifferentiate. In order to identify and trace mature osteogenic lineage cells, we have utilized transgenic mouse models in which the dentin matrix protein 1 (Dmp1) promoter drives expression of GFP (active marker) or Cre recombinase (historic label) in preosteocytes/osteocytes. In long bone chip outgrowth cultures, in which cells on the bone surface were enzymatically removed, cells with previous activity of the Dmp1 promoter migrated onto plastic and down-regulated Dmp1-GFP expression. Dmp1Cre-labeled cells from these cultures had the potential to re-differentiate into the osteogenic lineage, while the negative population showed evidence of adipogenesis. We observed numerous Dmp1Cre-labeled osteoblasts on the surface of bone chips following their *in vivo* transplantation. Our data indicate that cells embedded in bone matrix are motile, and once given access to the extra bony milieu will migrate out of their lacunae. This population of cells is phenotypically and functionally heterogeneous *in vitro*. Once the preosteocytes/osteocytes leave lacunae, they can dedifferentiate, potentially providing an additional source of functional osteoblasts.

## Introduction

Osteocytes, the cells residing within the bone matrix, represent more than 95% of the cellular component of adult bone tissue. They communicate with other osteocytes, and osteoblast lineage cells at the endosteal surface through the lacuna-canalicular system. Formation of this complex network is ideally suited for mechanosensation and integration of local and systemic signals. Osteocytes originate from mesenchymal stem cells (MSCs) through osteoblast lineage differentiation, with only 10–20% of osteoblasts differentiating into osteocytes [1]. Although there are still many unanswered questions about the differentiation process, our knowledge of the transition from osteoblast to osteocyte has expanded dramatically due to the identification of several osteocyte specific markers such as dentin matrix protein 1 (Dmp1) [2], [3], [4], [5], matrix extracellular phosphoglycoprotein (MEPE), phosphate regulating endopeptidase homolog (PHEX) and sclerostin (Sost) [6], [7], [8]. Dmp1 is an extracellular matrix-associated phosphoprotein, with expression restricted to mineralized tissues in mouse, rat and chicken [2], [9]. Based on the expression of Dmp1 in osteocytes, several investigators have utilized different lengths of the Dmp1 promoter to direct Cre recombination to this cell type [10], [11], [12], [13].

Knowledge of the molecular signature of osteocytes has allowed a more direct analysis of the molecular and cellular biology of these cells. Insight into the process of osteoblast to osteocyte transition has been gained using transgenic mouse lines in which GFP variants are expressed in osteoblasts and/or osteocytes, allowing visual identification of cells at specific differentiation stages [5], [14]. In addition, the Cre/loxP system is a valuable technology to evaluate gene deletion or to target gene expression to specific tissues at a particular stage of differentiation. By combining cell-type specific Cre activity with a Cre-activated reporter gene, lineage tracing studies can be performed. Although the combinatorial approach of using visual markers for transgene expression and Cre/loxP recombination system has addressed some of the unresolved questions about the differentiation process, many aspects are still unknown.

Mature osteoblasts are one of the major cell types responsible for achieving a balance between bone resorption and the formation of new bone. Bone formation can be augmented by increased induction of mesenchymal progenitor cells into osteoprogenitors and their subsequent differentiation into osteoid-secreting osteoblasts. In addition, activation of quiescent bone lining cells into matrix-producing osteoblasts has been documented as a mechanism for increasing the bone forming cell population [15]. The lineage can also add to its overall activity by inhibition of apoptosis of osteoblasts and osteocytes. These processes are well documented by both *in vitro* and *in vivo* studies [16], [17], [18], [19], [20], [21]. However, the potential of the osteocyte, the most abundant cell in bone, to function as a bone-forming cell through its ability to dedifferentiate, has not been recognized.

Here, we evaluated the ability of Dmp1-expressing preosteocytes/osteocytes to dedifferentiate *in vitro* and *in vivo*. We have utilized transgenic mice in which the Dmp1 promoter drives GFP expression as a visual marker of the current stage of differentiation (Dmp1-GFP), or drives Cre recombinase in order to activate an historical reporter (Ai9) in osteocytes and their progeny (Dmp1Cre/Ai9). We have used a primary culture model in which long bone chips are enzymatically stripped of surface cells, then cultured, allowing outgrowth of cells. A similar model has been utilized to show the presence of MSCs within bone chips [22]. Here, we report that Dmp1Cre/Ai9 positive cells embedded in bone, can move out of their lacunae, and develop an osteoblastic phenotype *in vitro* and *in vivo*.

## Materials and Methods

### Ethics Statement

All animal protocols were approved by the Animal Care Committee of the University of Connecticut Health Center under protocol numbers 2009-584 and 100490-0815.

### Mice

Generation and genotyping of the 8 kb Dmp1 promoter-driven GFP (Dmp1-GFP) mice was described previously [5]. 10 kb Dmp1Cre mice were obtained from Dr. Feng [10], and bred with the Ai9 reporter mouse (stock # 007905, Jackson Labs, Bar Harbor, ME) to generate Dmp1Cre/Ai9 mice. The Ai9 mice harbor a targeted mutation of the *Gt*(ROSA)*26Sor* locus with a loxP-flanked STOP cassette preventing transcription of a CAG promoter-driven red fluorescent protein variant (tdTomato) [23]. Immunodeficient NOD/SCID/IL-2 receptor common gamma chain deficient mice (NOD/SCID/IL-2r null) were previously described [24].

### Bone outgrowth cell cultures (BOCs)

Femurs and tibias from 2-3-month-old mice were dissected and cleaned of adherent muscle and periosteum. The epiphyseal growth plates were removed and the bone marrow was flushed. The cortical bone was minced into chips, washed several times with PBS and digested in 0.25% trypsin-EDTA (Gibco, Life Technologies, Carlsbad, CA) supplemented with 0.2% collagenase A (Roche, Indianapolis, IN), for 45 minutes at 37°C with 300 rpm shaking. Chips were then washed thoroughly with PBS then either fixed for histology or plated at a density of bone chips from 2–3 mice per 100 mm dish in 6 ml of minimum essential media alpha (αMEM) containing 100 U/ml penicillin, 100 µg/ml streptomycin (Gibco) and 10% fetal calf serum (FCS, HyClone, Logan, UT) (basal culture conditions). On day 4, additional medium was added (4 ml), followed by a complete change on day 7. Chips were cultured for 10 days to allow the cells within the bone migrate out and adhere onto the culture dish.

### Histological analysis

Bone samples were fixed in 4% paraformaldehyde/PBS (pH 7.4) at 4°C for 3 days, decalcified in 14% EDTA for up to 7 days, placed in 30% sucrose overnight and embedded in cryomedium (Thermo Fisher Scientific, Waltham, MA). Sections of 5 µm were obtained using a Leica cryostat (Wetzler, Germany) and tape transfer system (Section-lab, Hiroshima, Japan). Images were obtained using appropriate filters optimized for green fluorescent protein (GFP) variants (Chroma Technology, Bellows Falls, VT) using an Observer Z1 microscope (Carl Zeiss, Thornwood, NY). Images were obtained in gray scale, pseudocolored and composite images were assembled. To obtain a full-size image of bone chip implants, images were scanned at high power and then stitched into a composite.

### Flow Cytometry

FACS phenotype analysis and cell sorting of Dmp1Cre/Ai9 cells were performed using BD FACSAria II or LSR II (BD Biosciences, San Jose, CA) equipped with five lasers and 18 fluorescence detectors. Following 10 days in culture, BOCs were digested with 0.25% trypsin and single-cell suspensions were prepared in staining medium (2% FCS in PBS). Sorting gates were defined using cells from non-transgenic mice. Dmp1Cre/Ai9^+^ and Dmp1Cre/Ai9^−^ populations were sorted in αMEM/20% FCS collection buffer kept at 4°C. For characterization of the cell surface phenotype, we used antibodies purchased from e-Biosciences (San Diego, CA) unless otherwise stated. The following antibodies were used: APC-anti-CD45 (clone 30-F11), biotin-anti-CD140aPDGFRα (clone APA5), FITC-anti-CD90.2/Thy-1.2 (clone 30-H12); FITC-anti-CD106 (clone 429); biotin-anti-CD105 (clone MJ7/18), efluor450 anti-CD31 (clone 390); Pacific blue anti-Sca-1 (clone D7) (BioLegend, San Diego, CA). Stainings with biotinylated antibodies underwent a secondary incubation with streptavidin coupled to efluor780. All data were analyzed using BD CellQuest or Diva software.

### *In vitro* studies

#### Differentiation assays

After one week of basal culture conditions, the BOC were cultured in αMEM/10% FCS, 50 µg/ml ascorbic acid, 8 mM β-glycerol phosphate for 2 weeks to induce osteogenic differentiation. To evaluate osteogenic differentiation in Dmp1Cre/Ai9^+^ and Dmp1Cre/Ai9^−^ sorted populations, cells were seeded as 20 µl spots containing 5×10^4^ cells [25]. After 2 h, αMEM/10% FCS was added, and 24 h later cells were placed under osteogenic induction. For adipogenic differentiation, cells were plated at a density of 1.3×10^4^ cells/cm^2^ in 24-well plates and induced using αMEM/10% FCS, 1 µM insulin, 0.5 µM rosiglitazone for 9 days.

#### Histochemical analysis of cell cultures

Osteogenic and adipogenic phenotypes were determined using modified von Kossa silver nitrate staining, and Oil Red O staining, respectively [14], [26]. Images were acquired using a flat bed scanner and processed with Adobe Photoshop.

#### Detection of epifluorescence

GFP expression in cell cultures was visualized using an Olympus IX50 inverted system microscope equipped with IX-FLA inverted reflected light fluorescence (Olympus America Inc., Melville, NY). A specific excitation wavelength was obtained using filters for GFPtopaz (excitation: D500/20, emission: D550/40) and RFP (excitation: 560/40, emission: 630/75). Images were recorded with a SPOT-camera (Diagnostic Instruments Inc., Sterling Heights, MI).

### Gene expression analysis

Cultures or bone chips were homogenized in Trizol solution using a polytron (Ika) for approximately 40 seconds. RNA was extracted and converted into cDNA utilizing Improm-II™ Reverse Transcription System (Promega). Real-time PCR was performed using TaqMan® Gene Expression Assays specific for Gapdh (Mm99999915_g1), dentin matrix protein 1 (Dmp1, Mm00803833­­_g1), bone sialoprotein (Bsp, Mm00492555_m1), Sost (Mm00470479_m1), adipsin (Mm00442664_m1) and adiponectin (Mm00456425_m1). Data obtained were normalized to Gapdh expression levels. Differences in expression between selected groups were compared using Student’s t-tests.

### Time lapse imaging

BOC cultures were examined with a Zeiss Observer.Z1 microscope (Carl Zeiss) equipped with an imaging chamber maintaining 37°C and 6% CO_2_ in a humidified atmosphere. Cells were imaged beginning 72 hours after initiation of the culture, for the successive 48 hours, and images were taken every 40 minutes using an AxioCam color digital camera controlled by a user-defined computation program [27]. Fluorescent expression of Dmp1Cre/Ai9 in cultures was examined using a TRITC red filter (Chroma). Final.zvi files were converted into tiff files using AxioVision digital image processing software (Carl Zeiss).

### Subcutaneous implantation of bone chips

Bone chips from two Dmp1Cre/Ai9 mice were prepared as described. Following enzymatic digestion and washes they were mixed with 200 µl 3 mg/ml collagen type I (BD Biosciences) prepared according to the manufacturer’s instructions [28]. Immunodeficient NOD/SCID/ILr-2 deficient mice (3–4 month old) were anesthetized with ketamine/xylazine (135 mg/kg and 15 mg/kg) and the bone chips/gel implant placed subcutaneously under the left and right scapula. After surgery, 0.08 mg/kg buprenorphine hydrochloride was administered subcutaneously for analgesia. Implants were performed as three separate experiments, with one mouse sacrificed after 5 days (total n = 3) and two after 28 days (total n = 6) in each experiment. Following x-ray imaging, implants and surrounding tissues were collected and processed for histological analysis.

## Results

### Expression patterns of the Dmp1 reporter genes

**Figure 1:**
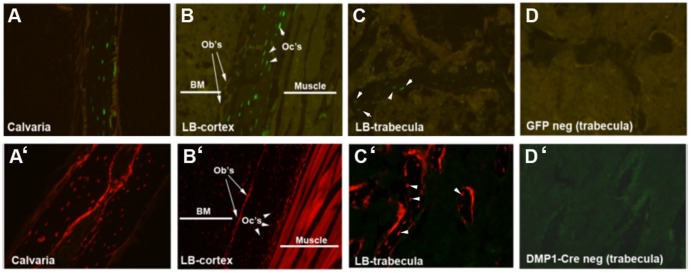
Histology of Dmp1-GFP and Dmp1Cre/Ai9 bones. Detection of GFP expression in Dmp1-GFP derived histological sections (A–D). A specific GFP signal was localized in osteocytes (arrowheads) of cortical bone in calvaria (A) and femur (B) and in trabeculae (C). Detection of tdTomato expression in histological sections derived from Dmp1Cre/Ai9 transgenic mice (A’–D’). Cre-directed recombination was detected in osteocytes (arrowheads) and in the osteoblast layer (arrows) of cortical bone from calvaria (A’) and femur (B’) and trabecular bone (C’). tdTomato expression was also present in skeletal muscle (B’). Sections derived from GFP negative (D) and Dmp1Cre negative (D’) mice served as controls. Images were taken at 10X magnification and are representative of histology performed on 6 mice of each genotype. BM, bone marrow; LB, long bone; Ob’s, osteoblasts; Oc’s, osteocytes.

In order to determine whether the Dmp1 reporters used for this study were osteocyte-specific, we first examined their distribution histologically. As described previously, Dmp1-GFP mice demonstrated restricted expression of GFP to cells embedded in the bone [5], [29]. Histological analysis of femur and calvaria from 2–3 month-old transgenic mice showed GFP signal in cortical and trabecular osteocytes (Fig. 1A–C). Sections of long bone trabeculae from non-transgenic mice were used to demonstrate the background fluorescence (Fig. 1D).

Dmp1Cre transgenic mice were crossed with the Ai9 reporter transgenic line to generate Dmp1Cre/Ai9 mice. In these compound mice, the expression of Cre is activated under the control of the 10kb Dmp1 promoter. Cre activity activates expression of the tdTomato visual reporter (Ai9), allowing lineage tracing of Dmp1-expressing cells and their progeny. Histological examination shows that Cre-directed recombination was detected in cortical osteocytes (indicated by arrowheads) and on some endocortical osteoblasts (indicated by arrows) (Fig. 1A’–B’). Similar expression of tdTomato was observed in the majority of osteocytes and some osteoblasts in trabecular bone (Fig. 1C’). The majority of skeletal muscle fibers were tdTomato positive indicating the activity of the promoter in the skeletal muscle or in the muscle progenitor cells at some point in the animal’s lifetime (Fig. 1B’).

### Dmp1Cre-labeled bone-embedded cells dedifferentiate *in vitro*

To evaluate the ability of preosteocytes/osteocytes to dedifferentiate *in vitro*, we established a bone outgrowth cell culture model. Histology of bone chips before and after the enzymatic digestion step confirmed that osteoblasts and bone lining cells were completely removed while osteocytes remained within the bone matrix (Fig. S1). After 3–5 days of culture, cells started to appear around the bone chips attached to the tissue culture plate, and migrate away from chips.

**Figure 2:**
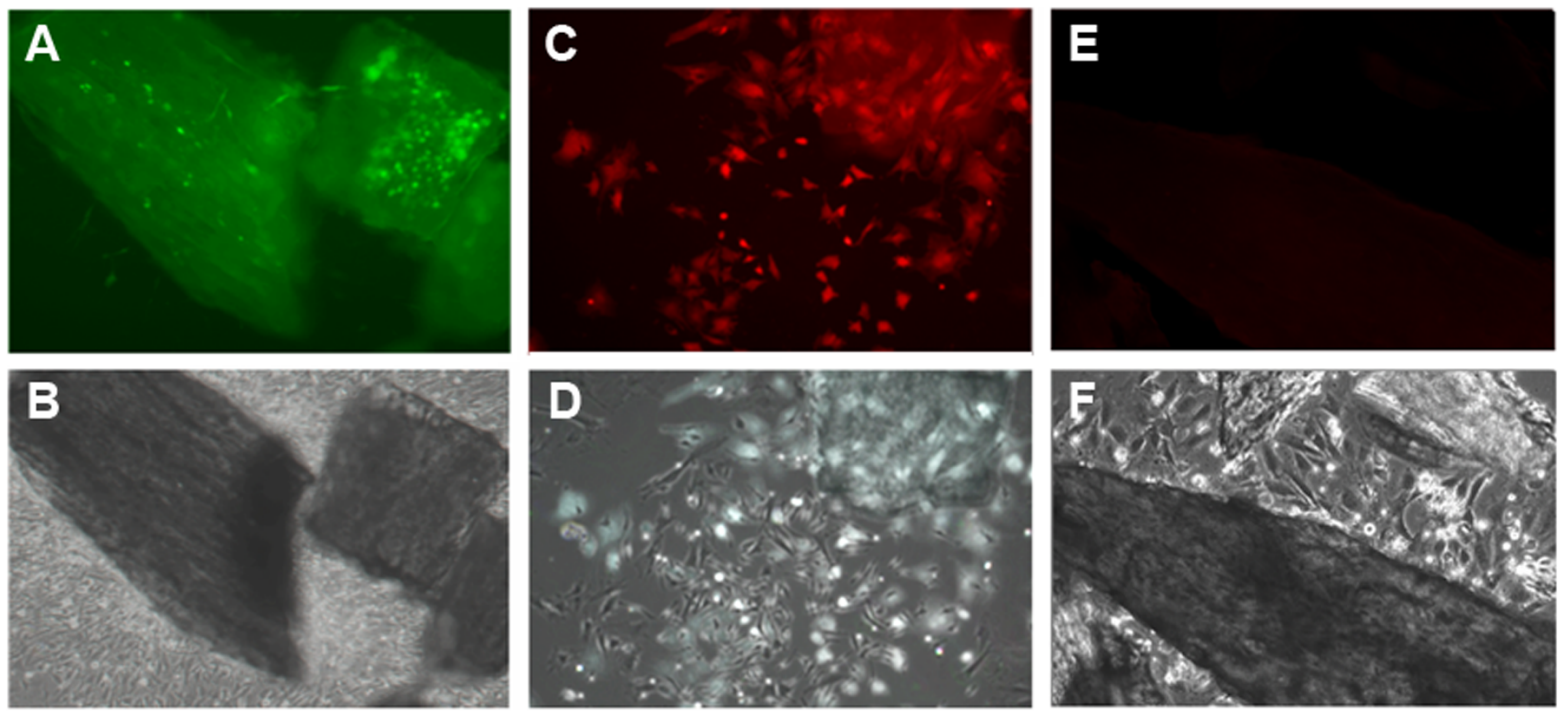
BOC are labeled by Dmp1Cre/Ai9 but do not express Dmp1-GFP. Primary bone chip outgrowth cell (BOC) cultures were obtained by enzymatic digestion of long bones derived from Dmp1-GFP (A–B) and Dmp1Cre/Ai9 transgenic mice (C–D). After 3–5 days of culture, cells started to crawl out of the chips (B, D, F). Dmp1-GFP expressing osteocytes can be observed within the bone chips while no GFP expression was observed in BOC by epifluorescence (A). BOC from Dmp1Cre/Ai9 chips showed tdTomato fluorescence in a small proportion of cells (C). None of the outgrowth cells derived from Dmp1Cre negative/Ai9 bone chips express Dmp1Cre/Ai9 (E). Upper panel, epifluorescence; lower panel, phase contrast imaging. Images were taken at 10X magnification and are representative of at least 5 independent cultures.

**Figure 3:**
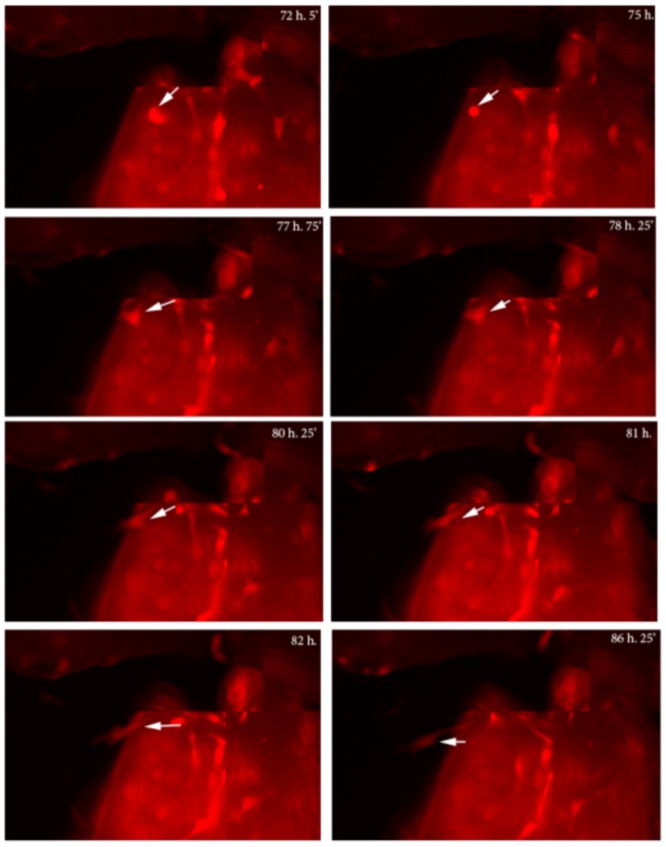
Time lapse imaging of Dmp1Cre/Ai9 derived bone chips. Bone chips were imaged every 40 minutes starting at day three of culture. The movement of Dmp1Cre^+^/Ai9 cells within the bone chips and their expulsion onto the culture plate was observed (moving cell is indicated by the arrow). Representative images spanning a 14 h 20 minute period from the time points indicated are shown. Imaging was performed on three independent cultures.

In cultures from Dmp1-GFP mice, GFP expression was restricted to osteocytes within the bone chips, while few or no cells expressing GFP were observed in BOCs by epifluorescence (Fig. 2A–B), or by immunofluorescent staining for GFP (data not shown). These data indicate that at this point in culture, BOC do not show detectable activity of the osteocyte-specific Dmp1 promoter. To determine whether the BOCs are the progeny of osteocytes, cultures were performed using bones from Dmp1Cre/Ai9 mice. In contrast to the absence of Dmp1-GFP expression (Fig. 2A–B), BOCs from Dmp1Cre/Ai9 mice showed tdTomato fluorescence in a numerous cells surrounding bone chips (Fig. 2C–D). As tdTomato is expressed only in cells in which Cre was once active, and Dmp1 promoter activity is absent in the BOCs, these data confirm that tdTomato expression is due to the historical activation of the Ai9 transgene by Dmp1Cre. To confirm that Dmp1Cre/Ai9^+^ BOCs were derived from Dmp1Cre/Ai9^+^ bone embedded cells, we performed time lapse imaging. Bone chips derived from Dmp1Cre/Ai9 mice were visualized for 48 hours, starting at day three of culture. We observed motile Dmp1Cre/Ai9^+^ cells migrate towards the edge of bone chips, then onto the surface of the culture dish, moving away from the bone chip in the following hours (Fig. 3, Movie S1).

### Phenotypical characterization of Dmp1Cre/Ai9^+^ cells

**Figure 4:**
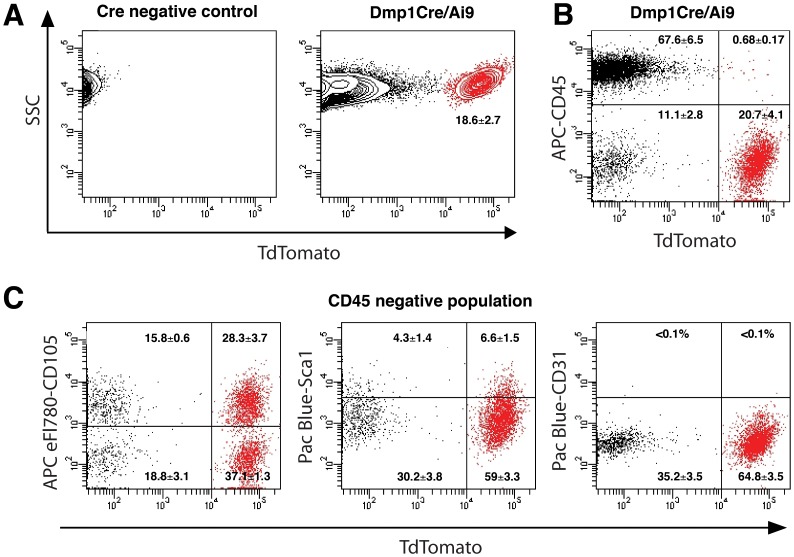
Cell surface marker expression in DmpCre/Ai9^+^ and DmpCre/Ai9^−^ populations of BOC cultures. Primary BOC isolated from Dmp1Cre/Ai9 mice were grown for ten days before analysis. Nontransgenic cells were utilized as controls to set the gate for tdTomato^+^ population (A, mean±SEM, n = 8). Expression of pan-hematopoietic marker CD45 was evaluated in the BOC population (B). Expression of CD105, Sca1, and endothelial marker CD31 was evaluated in the CD45^−^ BOC cells (C). Dot-plots from a representative experiment are shown. Percentages within gated populations represent mean±SEM of four biological replicates. Lineage marker expression was analyzed on at least 10^4^ gated cells. Gates were set in accord to the non-stained sample (not shown).

Previous work by Zhu et al. indicated that cells derived from bone chips are mesenchymal progenitors capable of multi-lineage differentiation [22]. To investigate whether the Dmp1Cre/Ai9^+^ BOCs showed characteristics of mesenchymal progenitor cells, we analyzed the cell surface phenotype by flow cytometry. FACS analysis indicated that the Dmp1Cre-activated transgene is present in 18.6±2.7% (mean±SEM, n = 8) of cells (Fig. 4A and Fig. S2B). We evaluated the expression of Dmp1Cre/Ai9^+^ cells for the presence of CD45, a pan hematopoietic marker. Over 95% of Dmp1Cre/Ai9^+^ cells were CD45^−^ (96.1±1.6%), while 85.3±4.1% of the Dmp1Cre/Ai9^−^ population was CD45^+^ (Fig. 4B). We also analyzed the CD45^−^ compartment of Dmp1Cre/Ai9^+^ and Dmp1Cre/Ai9^−^ populations for the presence of mesenchymal and endothelial markers. BOCs that are Dmp1Cre/Ai9^+^/CD45^−^ were negative for the endothelial marker CD31 (Fig. 4C) as well as for mesenchymal markers CD106, CD90 and CD140a/PDGFRα (data not shown). We also observed expression of CD105 on 42.8±3.6%, while Sca1 was expressed in a small population of Dmp1Cre/Ai9^+^/CD45^−^ cells (9.9±2.2%) (Figure 4C). Our data suggest that cells derived from bone represent a heterogeneous population with only a small proportion of them expressing markers associated with mesenchymal stem/progenitor cells.

### Differentiation potential of Dmp1Cre/Ai9^+^ BOCs *in vitro*

**Figure 5:**
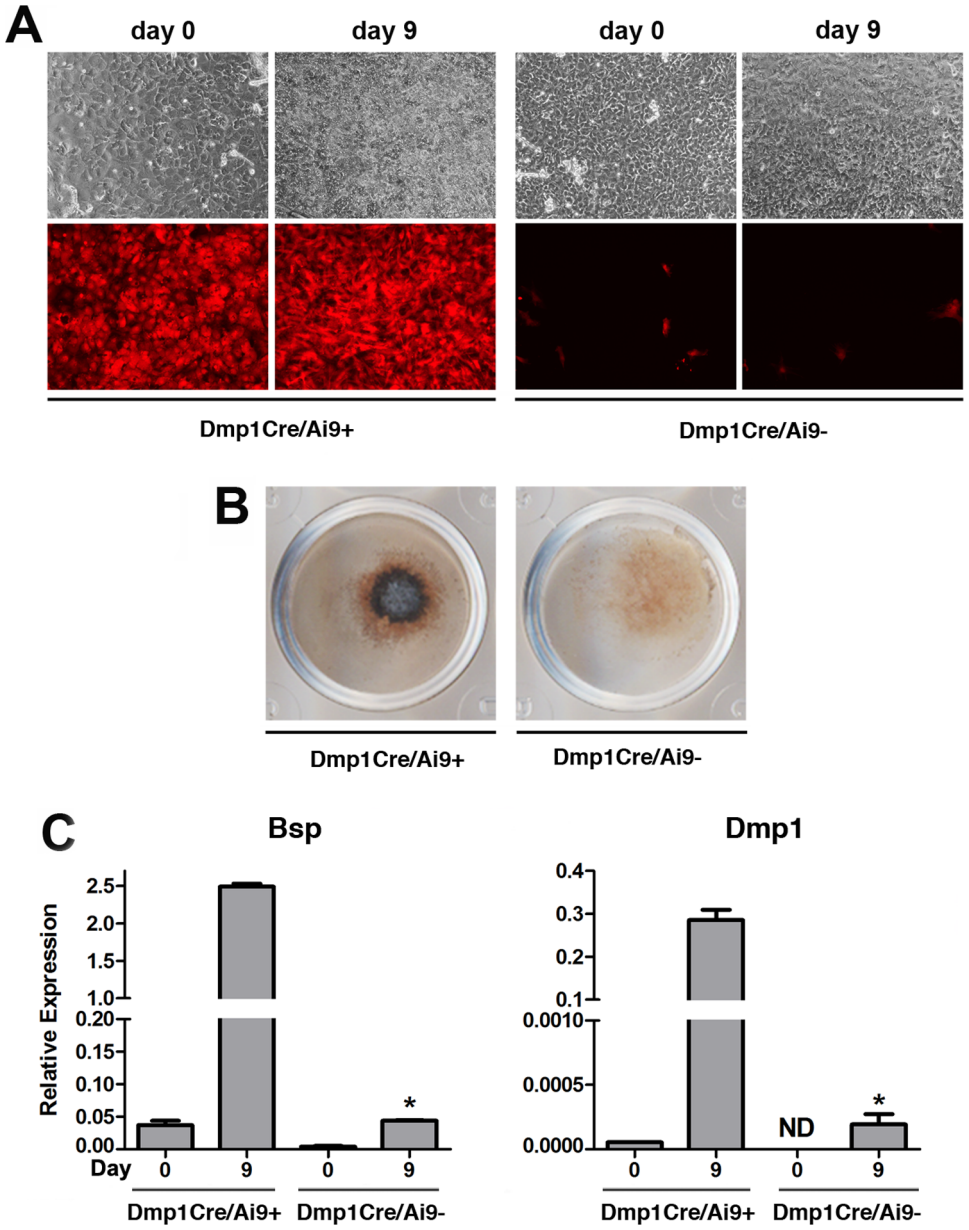
Osteogenic potential of BOCs. Following sorting, Dmp1Cre/Ai9^+^ and Dmp1Cre/Ai9^−^ populations were cultured under basal conditions overnight (indicated as day 0) before treatment with osteogenic medium. After 9 days of osteogenic induction, Dmp1Cre/Ai9^+^ cells underwent mineralization (A). Osteogenic differentiation was confirmed by von Kossa staining (B) and real-time PCR analysis of Bsp and Dmp1 mRNA expression (C). Real-time PCR results are presented as mean±SEM of data normalized to Gapdh expression and pooled from two biological replicates. * P<0.05 compared to Dmp1Cre/Ai9^+^ day 9 as determined by two-tailed Student’s t-test. Bsp, bone sialoprotein; Dmp1, dentin matrix protein 1; ND, not detectable.

**Figure 6:**
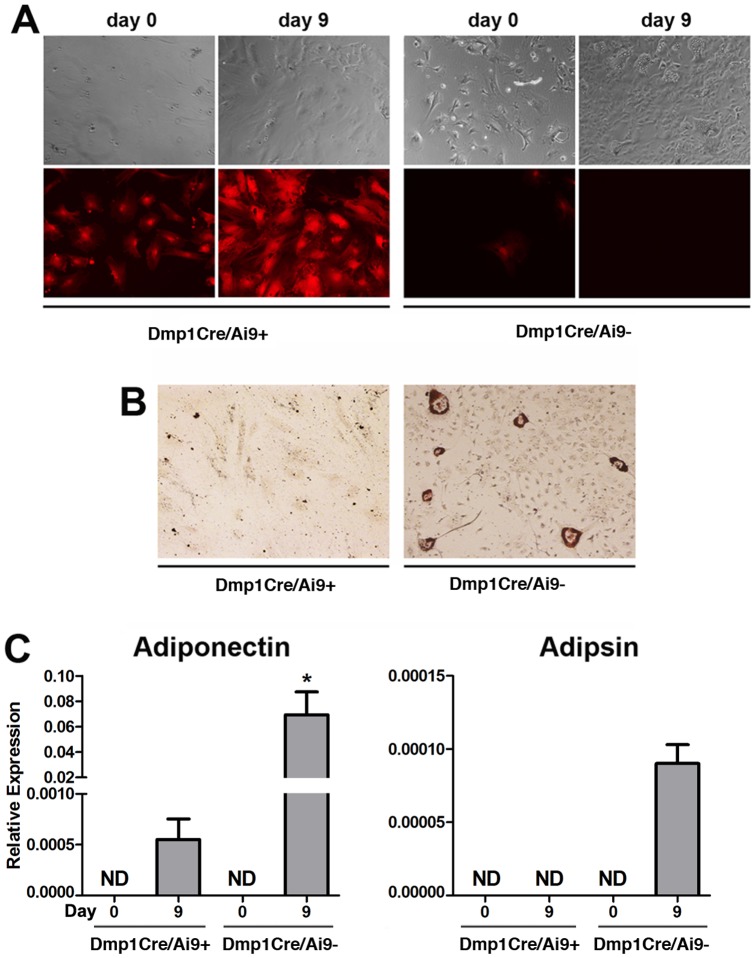
Adipogenic potential of BOCs. Sorted Dmp1Cre/Ai9^+^ and Dmp1Cre/Ai9^−^ cells were cultured under basal conditions for 3 days (indicated as day 0), then treated with adipogenic medium. Unlike Dmp1Cre/Ai9^+^, Dmp1Cre/Ai9^−^ cells showed evidence of adipogenesis (A). Adipogenic differentiation was assessed by Oil Red O staining (B) and adiponectin and adipsin expression levels by real-time PCR analysis (C). Real-time PCR results are presented as mean±SEM of data normalized to Gapdh expression and pooled from two biological replicates. * P<0.05 compared to Dmp1Cre/Ai9^+^ day 9 as determined by two-tailed Student’s t-test. Statistical analysis was only performed where expression was detectable in both samples. ND, not detectable.

To test the extent of dedifferentiation and mesenchymal lineage differentiation potential of cells from BOC cultures, we sorted Dmp1Cre/Ai9^+^ and Dmp1Cre/Ai9^−^ populations (Fig S2A–D). Gene expression analysis of cells immediately post sorting indicated that Dmp1Cre/Ai9^+^ and Dmp1Cre/Ai9^−^ populations both showed detectable Dmp1 expression, although levels were consistently lower than in digested bone chips which are enriched for mature osteocytes (Fig. S2E). In addition, the marker of mature osteocytes, Sost, was not expressed in Dmp1Cre/Ai9^+^ and Dmp1Cre/Ai9^−^ cells (Fig. S2F). To evaluate their *in vitro* differentiation potential, sorted BOCs were grown under osteogenic and adipogenic conditions. After 9 days of osteogenic induction, Dmp1Cre/Ai9^+^ cells formed mineralized matrix indicated by von Kossa staining while Dmp1Cre/Ai9^−^ cells did not exhibit that potential (Fig. 5A–B). The mature osteoblast/osteocyte phenotype of cells derived from the Dmp1Cre/Ai9^+^ population was confirmed by induction of Bsp and Dmp1 expression (Fig. 5C). To substantiate these results, we established primary BOC cultures from Dmp1Cre/Ai9/Dmp1-GFP transgenic mice. After one week in culture, GFP fluorescence was restricted to osteocytes within the bone chips, while tdTomato was evident both in cells embedded in the bone and in BOCs (Fig. S3). After 6 days of osteogenic induction, a few Dmp1Cre/Ai9^+^ cells became Dmp1-GFP^+^ and by 14 days a higher proportion of Dmp1Cre/Ai9^+^ cells coexpressed Dmp1-GFP^+^ and formed mineralized nodules (Fig. S3). We also tested the ability of sorted cells to undergo adipogenesis. Adipocytes formed in the Dmp1Cre/Ai9^−^ cultures, but not the Dmp1Cre/Ai9^+^ cultures (Fig. 6A–C).

### Dmp1Cre/Ai9^+^ cells can dedifferentiate *in vivo*

**Figure 7:**
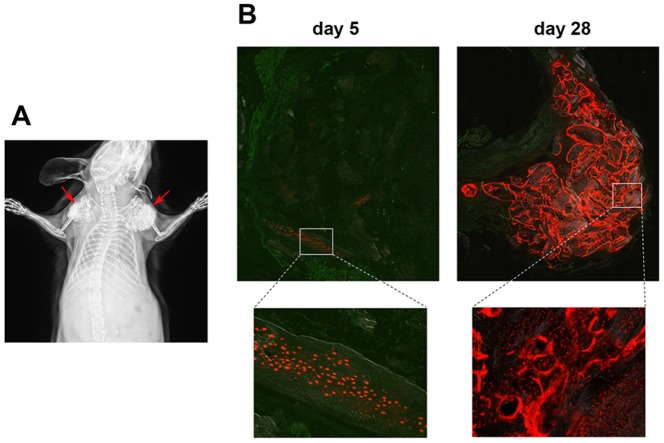
Dmp1Cre/Ai9^+^ cells dedifferentiate *in vivo.* Digested bone chips derived from Dmp1Cre/Ai9 mice were mixed with collagen type I and implanted subcutaneously in immunodeficient mice, as shown by the representative x-ray image (A**,** arrows). Five and twenty-eight days following implantation, the distribution of tdTomato^+^ cells was determined by histology (B).

To test whether the Dmp1Cre/Ai9^+^ preosteocyte/osteocytes are able to generate osteoblasts *in vivo* we traced Dmp1Cre/Ai9^+^ cells in a subcutaneous implantation model. Bone chips derived from Dmp1Cre/Ai9 mice were enzymatically digested to removed bone surface cells, mixed with collagen gel, and implanted subcutaneously in immunodeficient mice, as shown by x-ray (Fig. 7A). Five days after implantation, tdTomato expression was still restricted to cells embedded within bone (Fig. 7B), as we observed immediately following enzymatic digestion (Fig. S1). Four weeks later, numerous Dmp1Cre/Ai9^+^ osteoblasts were observed on bone chip surfaces. These data indicate that preosteocytes/osteocytes can migrate out of bone and onto bone surfaces, dedifferentiating to osteoblasts. These data provide an *in vivo* correlate to our *in vitro* data.

## Discussion

Cell dedifferentiation has been observed in a variety of biological phenomena, such as cancer, organ regeneration, and stem cell renewal, but it has been difficult to study due to a lack of traceable experimental systems**.** In some tissues, cells that have already begun to specialize can revert or "dedifferentiate" and assume stem cell properties, including the ability to self-renew. Dedifferentiation of specialized cells could provide a "reservoir" of cells that may act as an additional resource that can be utilized in extreme conditions such as injury repair that require massive lineage activation [30], [31]. Extensive evidence of dedifferentiation exists in amphibians such as axolotls, where all tissue types, including muscle, dermis, spinal cord, and cartilage regrow during tail regeneration. Regeneration potential in mammals is much more limited, but digit tips in mice and humans can regenerate. Lineage tracing in mice after digit tip amputation indicated that tissues in the regenerated digit including the dermis, bone, tendon and vasculature were derived from local, lineage-restricted progenitors, rather than a pluripotent stem cell or circulating population [32]. The Cre transgenes used to label cells in this study were not progenitor-specific, so a role for cellular dedifferentiation in the provision of lineage-restricted progenitors in this process cannot be ruled out.

Dedifferentiation of mammalian cells has been demonstrated *in vitro*. Ectopic expression of *Msx1* in C2C12 myotubes down-regulated the nuclear muscle proteins MyoD and myogenin and some myotubes cleave to produce smaller myotubes or proliferating, mononucleated cells [33]. Following these dedifferentiation steps, clonal populations of the myotube-derived mononucleated cells can be induced to re-differentiate into cells expressing chondrogenic, adipogenic, myogenic, and osteogenic markers, suggesting that terminally differentiated myotubes can dedifferentiate into multipotent progenitors when stimulated with the appropriate signals [33]. Mature adipocytes are also capable of *in vitro* dedifferentiation using a method known as ceiling culture [34]. The fibroblastic cell population generated, known as DFAT cells, can re-differentiate to multiple lineages including adipocytes, osteoblasts and chondrocytes *in vitro*, and reconstitute adipose tissue, including vascular elements, *in vivo* [35], [36]. Human DFAT cells have also been reported to show other MSC-like properties including support of hematopoiesis [37]. It is not clear, however, whether this process can occur *in vivo*. Transdifferentiation of human MSC-derived osteoblasts, chondrocytes and adipocytes to alternative lineages has also been demonstrated after replating of differentiated cultures *in vitro*, although in most of these cultures only a subset of the replated cells would have reached terminal differentiation stages [38].

Presently, there is no evidence that osteocytes, which are considered terminally differentiated cells, are capable of dedifferentiation. Dedifferentiation of preosteocytes/osteocytes could provide an unappreciated source of osteoblasts capable of proliferation, differentiation and bone formation when bone is under extreme stress. In our study, we have presented evidence demonstrating the ability of preosteocytes/osteocytes to undergo dedifferentiation *in vitro* and *in vivo*. This process generated cells that could re-differentiate into bone matrix-producing osteoblasts. We have utilized transgenic mice in which the Dmp1 promoter drives the expression of Cre recombinase [10]. One limitation of this model was the presence of Cre recombinase activity in osteoblasts on the bone surface. However, following enzymatic digestion of the bone chips we were able to remove all cells, including osteoblasts, from the bone surface and proceed to culture chips where all Dmp1Cre/Ai9^+^ cells were matrix-embedded (Fig. S1). Time-lapse imaging confirmed the movement of cells within the chips, and their subsequent ability to migrate out of the bone and adhere to tissue culture plastic. Similar experiments describing cell movement within live calvaria have been reported [39], [40]. Veno et al. have also shown the presence of a rare Dmp1-GFP^+^ cell population on the surface of calvaria that can become very motile, move and then embed within the matrix [41].

Zhu et al. have described a similar protocol to the one used in this study for culturing enzymatically-digested mouse long bone chips in order to isolate MSCs rapidly and with minimal contamination [22]. However, we postulated that the primary culture was heterogeneous, and included a population of cells that in their lifetime expressed the Dmp1 promoter. Our cultures appear to produce cells more slowly than the previous report [22], possibly due to the use of older animals. Our cultures also show much more varied cell surface marker expression, with extensive contamination of cells expressing hematopoietic cell markers. While the enzymatic digestion removes bone surface cells, the intracortical microvasculature and innervation is not eliminated, providing a potential source of hematopoietic cells, as well as other cell types such as MSCs. It is notable that Zhu et al. do not assess the cell surface marker phenotype or perform differentiation assays until cultures have been passaged three times. This allows expansion of stem/progenitor cells, and dilution of hematopoietic contaminants and preosteocyte/osteocyte-derived cells, which are likely to have more limited proliferative potential. Characterization of the Dmp1Cre/Ai9^+^ population indicated that they do not express MSC markers such as CD106, CD90 or PDGFRα. In addition to lacking Dmp1-GFP expression, they show loss of mature osteocyte marker Sost, and greatly reduced endogenous Dmp1 expression compared to bone chips. Observations of cells after replating suggest they also have some proliferative capacity, although this capacity was not directly tested in this study. These cells were able to differentiate into mature osteoblasts and following mineralization, activate the Dmp1-GFP reporter, but their differentiation potential appears to be restricted to the osteogenic lineage. However, one of the MSC markers examined, CD105, was expressed in a high proportion of cells. Sorting cells based on CD105 expression *ex vivo* produces a population of cells enriched for MSC-like properties, and it is expressed uniformly in MSC cultures, but its expression is not unique to MSCs [42], [43]. Gene expression profiling of differentiating calvarial osteoblast cultures has indicated that, while expression of MSC markers such as Sca1, CD106 and CD90 are down-regulated in differentiated osteoblasts, CD105 expression does not change, suggesting that in the *in vitro* context it can be expressed in more mature osteoblast lineage cells [44]. Sca1, a widely used marker of mouse MSCs [43], [45], was expressed on a small proportion of CD45^−^ cells in both the Dmp1Cre/Ai9^+^ and Dmp1Cre/Ai9^−^ fractions, suggesting there may be a subset of immature mesenchymal progenitors in the cultures. The Dmp1Cre/Ai9^−^ population showed restricted differentiation potential, showing some adipogenesis, but not osteogenic differentiation, and heterogeneous cell surface marker expression. These observations confirm the heterogeneity of the cells that were mobilized from bone chips, and suggest that cells that formerly demonstrated a preosteocyte/osteocyte-like phenotype do not undergo complete dedifferentiation to a multi-potent cell type, but contribute to a mixed population with osteogenic potential.

It is well established that *in vitro* culture of cells can alter their phenotype, and plasticity [42], [46]. We therefore sought to assess the potential for osteocyte dedifferentiation *in vivo*. However, *in vivo* lineage tracing studies in clinically relevant models such as fracture healing were precluded by Dmp1Cre activity in osteoblasts. To circumvent this, we implanted denuded bone chips derived from Dmp1Cre/Ai9 mice subcutaneously into immunodeficient mice. Following four weeks *in vivo*, numerous osteoblasts were observed lining the bone surfaces. This observation indicates that the Dmp1Cre/Ai9^+^ cells from the donor animal have the ability to dedifferentiate *in vivo*. Future studies using more osteocyte-specific labeling systems, such as the inducible Dmp1CreERT2 [13] should be utilized in future to confirm these results.

In summary, we have shown that bone-embedded preosteocytes/osteocytes have the ability to dedifferentiate into less mature osteogenic cells. Given that preosteocytes/osteocytes are embedded within mineralized tissue *in vivo*, dedifferentiation would likely require the ability to sense and gain access to the extra-bony milieu. Fracture, joint replacement, and placement of screws in the bone for securing plates and rods all expose the inner bone to the extra-bony space. We can only speculate that this exposure may mobilize osteocytes to dedifferentiate and then re-differentiate, allowing them to participate in the repair process. Methods of labeling osteocytes more specifically are required to demonstrate this experimentally. Understanding the mechanisms by which mature osteogenic cells activate their dedifferentiation potential, and how these programs are utilized during tissue repair could provide powerful strategies for regenerative medicine.

## Supporting Information

Figure S1 Histological analysis of Dmp1Cre/Ai9 derived bone chips. Bones derived from Dmp1Cre/Ai9 mice were minced into <1 mm chips. Histology of bone chips before
(A) and after (B) enzymatic digest to remove cells on the bone surface was performed. Following digestion, osteoblasts (arrows) and lining cells were completely removed while osteocytes (arrowheads) remained within the bone matrix. Images were taken at 10X magnification. BM, bone marrow; Ob’s, osteoblasts; Oc’s, osteocytes.
(TIF)

Figure S2 Cell sorting of BOC cultures derived from Dmp1Cre/Ai9 mice. BOC from Dmp1Cre/Ai9 mice were cultured for before FACS sorting. Nontransgenic cells were utilized as controls to preset the sorting gates (A). The proportion of Dmp1Cre/Ai9^+^ and Dmp1Cre/Ai9^−^ cells is indicated (B). Reanalysis of the sorted population purity is shown for Dmp1Cre/Ai9^−^ (C) and Dmp1Cre/Ai9^+^ (D) cells. Data is representative of five different sorting experiments. RNA from digested bone chips (n = 2), and from 10 day Dmp1Cre/Ai9^+^ and Dmp1Cre/Ai9^−^ cultured cells immediately post sorting (n = 3) was used to assess expression of osteocyte marker genes Dmp1 (E) and Sost (F). Expression levels are presented as mean±SEM and are normalized to Gapdh expression. Changes in Dmp1 expression were not statistically significant. ND, not detectable; SSC, side scatter.
(TIF)

Figure S3 Differentiation of Dmp1Cre/Ai9^+^ BOC into Dmp1-GFP expressing mature osteoblast cells. Primary BOC cultures from Dmp1Cre/Ai9/Dmp1-GFP transgenic mice were cultured under basal conditions for 7 days (indicated as day 0) before treatment with osteogenic medium for 6 or 14 days. Osteogenic differentiation was confirmed by dual expression of red-labeled Dmp1Cre/Ai9 and green-labeled Dmp1-GFP cells in conjunction with the formation of mineralized nodules. BF, brightfield.
(TIF)

Movie S1 Time lapse imaging of Dmp1Cre/Ai9 derived bone chips. Bone chips were imaged every 40 minutes for 48 hours starting at day three of culture. The movement of Dmp1Cre^+^/Ai9 cells within the bone chips and their expulsion onto the culture plate was observed.
(mp4)

## References

[1] Aubin JE, Turksen K (1996) Monoclonal antibodies as tools for studying the osteoblast lineage. Microscopy research and technique 33: 128–140. 8845513 10.1002/(SICI)1097-0029(19960201)33:2<128::AID-JEMT4>3.0.CO;2-P

[2] Toyosawa S, Shintani S, Fujiwara T, Ooshima T, Sato A, et al (2001) Dentin matrix protein 1 is predominantly expressed in chicken and rat osteocytes but not in osteoblasts. Journal of bone and mineral research : the official journal of the American Society for Bone and Mineral Research 16: 2017–2026. 10.1359/jbmr.2001.16.11.2017 11697797

[3] Feng JQ, Ward LM, Liu S, Lu Y, Xie Y, et al (2006) Loss of DMP1 causes rickets and osteomalacia and identifies a role for osteocytes in mineral metabolism. Nature genetics 38: 1310–1315. 17033621 10.1038/ng1905 PMC1839871

[4] He G, George A (2004) Dentin matrix protein 1 immobilized on type I collagen fibrils facilitates apatite deposition in vitro. The Journal of biological chemistry 279: 11649–11656. 14699165 10.1074/jbc.M309296200

[5] Kalajzic I, Braut A, Guo D, Jiang X, Kronenberg MS, et al (2004) Dentin matrix protein 1 expression during osteoblastic differentiation, generation of an osteocyte GFP-transgene. Bone 35: 74–82. 15207743 10.1016/j.bone.2004.03.006

[6] Fisher LW, Fedarko NS (2003) Six genes expressed in bones and teeth encode the current members of the SIBLING family of proteins. Connective tissue research 44 Suppl 133–40. 12952171

[7] Ruchon AF, Tenenhouse HS, Marcinkiewicz M, Siegfried G, Aubin JE, et al (2000) Developmental expression and tissue distribution of Phex protein: effect of the Hyp mutation and relationship to bone markers. Journal of bone and mineral research : the official journal of the American Society for Bone and Mineral Research 15: 1440–1450. 10.1359/jbmr.2000.15.8.1440 10934642

[8] Poole KE, van Bezooijen RL, Loveridge N, Hamersma H, Papapoulos SE, et al (2005) Sclerostin is a delayed secreted product of osteocytes that inhibits bone formation. FASEB journal : official publication of the Federation of American Societies for Experimental Biology 19: 1842–1844. 16123173 10.1096/fj.05-4221fje

[9] Feng JQ, Huang H, Lu Y, Ye L, Xie Y, et al (2003) The Dentin matrix protein 1 (Dmp1) is specifically expressed in mineralized, but not soft, tissues during development. Journal of dental research 82: 776–780. 14514755 10.1177/154405910308201003

[10] Lu Y, Xie Y, Zhang S, Dusevich V, Bonewald LF, et al (2007) DMP1-targeted Cre expression in odontoblasts and osteocytes. J Dent Res 86: 320–325. 17384025 10.1177/154405910708600404

[11] Xiao Z, Dallas M, Qiu N, Nicolella D, Cao L, et al (2011) Conditional deletion of Pkd1 in osteocytes disrupts skeletal mechanosensing in mice. FASEB J 25: 2418–2432. 21454365 10.1096/fj.10-180299 PMC3219213

[12] Kramer I, Halleux C, Keller H, Pegurri M, Gooi JH, et al (2010) Osteocyte Wnt/beta-catenin signaling is required for normal bone homeostasis. Mol Cell Biol 30: 3071–3085. 20404086 10.1128/MCB.01428-09 PMC2876685

[13] Powell WF Jr, Barry KJ, Tulum I, Kobayashi T, Harris SE, et al (2011) Targeted ablation of the PTH/PTHrP receptor in osteocytes impairs bone structure and homeostatic calcemic responses. J Endocrinol 209: 21–32. 21220409 10.1530/JOE-10-0308 PMC3783949

[14] Kalajzic I, Kalajzic Z, Kaliterna M, Gronowicz G, Clark SH, et al (2002) Use of type I collagen green fluorescent protein transgenes to identify subpopulations of cells at different stages of the osteoblast lineage. Journal of bone and mineral research : the official journal of the American Society for Bone and Mineral Research 17: 15–25. 10.1359/jbmr.2002.17.1.15 11771662

[15] Jilka RL (2007) Molecular and cellular mechanisms of the anabolic effect of intermittent PTH. Bone 40: 1434–1446. 17517365 10.1016/j.bone.2007.03.017 PMC1995599

[16] Jilka RL, Weinstein RS, Bellido T, Parfitt AM, Manolagas SC (1998) Osteoblast programmed cell death (apoptosis): modulation by growth factors and cytokines. Journal of bone and mineral research : the official journal of the American Society for Bone and Mineral Research 13: 793–802. 10.1359/jbmr.1998.13.5.793 9610743

[17] Jilka RL, Weinstein RS, Bellido T, Roberson P, Parfitt AM, et al (1999) Increased bone formation by prevention of osteoblast apoptosis with parathyroid hormone. The Journal of clinical investigation 104: 439–446. 10449436 10.1172/JCI6610 PMC408524

[18] Plotkin LI, Manolagas SC, Bellido T (2002) Transduction of cell survival signals by connexin-43 hemichannels. The Journal of biological chemistry 277: 8648–8657. 11741942 10.1074/jbc.M108625200

[19] Plotkin LI, Manolagas SC, Bellido T (2007) Glucocorticoids induce osteocyte apoptosis by blocking focal adhesion kinase-mediated survival. Evidence for inside-out signaling leading to anoikis. The Journal of biological chemistry 282: 24120–24130. 17581824 10.1074/jbc.M611435200

[20] Plotkin LI, Mathov I, Aguirre JI, Parfitt AM, Manolagas SC, et al (2005) Mechanical stimulation prevents osteocyte apoptosis: requirement of integrins, Src kinases, and ERKs. American journal of physiology Cell physiology 289: C633–643. 15872009 10.1152/ajpcell.00278.2004

[21] Plotkin LI, Weinstein RS, Parfitt AM, Roberson PK, Manolagas SC, et al (1999) Prevention of osteocyte and osteoblast apoptosis by bisphosphonates and calcitonin. The Journal of clinical investigation 104: 1363–1374. 10562298 10.1172/JCI6800 PMC409837

[22] Zhu H, Guo ZK, Jiang XX, Li H, Wang XY, et al (2010) A protocol for isolation and culture of mesenchymal stem cells from mouse compact bone. Nature protocols 5: 550–560. 20203670 10.1038/nprot.2009.238

[23] Madisen L, Zwingman TA, Sunkin SM, Oh SW, Zariwala HA, et al (2010) A robust and high-throughput Cre reporting and characterization system for the whole mouse brain. Nat Neurosci 13: 133–140. 20023653 10.1038/nn.2467 PMC2840225

[24] Ishikawa F, Yasukawa M, Lyons B, Yoshida S, Miyamoto T, et al (2005) Development of functional human blood and immune systems in NOD/SCID/IL2 receptor {gamma} chain(null) mice. Blood 106: 1565–1573. 15920010 10.1182/blood-2005-02-0516 PMC1895228

[25] Liu Y, Wang L, Fatahi R, Kronenberg M, Kalajzic I, et al (2010) Isolation of murine bone marrow derived mesenchymal stem cells using Twist2 Cre transgenic mice. Bone 47: 916–925. 20673822 10.1016/j.bone.2010.07.022 PMC2952694

[26] Kalajzic Z, Li H, Wang LP, Jiang X, Lamothe K, et al (2008) Use of an alpha-smooth muscle actin GFP reporter to identify an osteoprogenitor population. Bone 43: 501–510. 18571490 10.1016/j.bone.2008.04.023 PMC2614133

[27] Wang YH, Liu Y, Buhl K, Rowe DW (2005) Comparison of the action of transient and continuous PTH on primary osteoblast cultures expressing differentiation stage-specific GFP. Journal of bone and mineral research : the official journal of the American Society for Bone and Mineral Research 20: 5–14. 10.1359/JBMR.041016 15619664

[28] Rutherford RB, Moalli M, Franceschi RT, Wang D, Gu K, et al (2002) Bone morphogenetic protein-transduced human fibroblasts convert to osteoblasts and form bone in vivo. Tissue engineering 8: 441–452. 12167230 10.1089/107632702760184709

[29] Yang W, Lu Y, Kalajzic I, Guo D, Harris MA, et al (2005) Dentin matrix protein 1 gene cis-regulation: use in osteocytes to characterize local responses to mechanical loading in vitro and in vivo. J Biol Chem 280: 20680–20690. 15728181 10.1074/jbc.M500104200

[30] Echeverri K, Clarke JD, Tanaka EM (2001) In vivo imaging indicates muscle fiber dedifferentiation is a major contributor to the regenerating tail blastema. Dev Biol 236: 151–164. 11456451 10.1006/dbio.2001.0312

[31] Lo DC, Allen F, Brockes JP (1993) Reversal of muscle differentiation during urodele limb regeneration. Proc Natl Acad Sci U S A 90: 7230–7234. 8346239 10.1073/pnas.90.15.7230 PMC47110

[32] Rinkevich Y, Lindau P, Ueno H, Longaker MT, Weissman IL (2011) Germ-layer and lineage-restricted stem/progenitors regenerate the mouse digit tip. Nature 476: 409–413. 21866153 10.1038/nature10346 PMC3812235

[33] Odelberg SJ, Kollhoff A, Keating MT (2000) Dedifferentiation of mammalian myotubes induced by msx1. Cell 103: 1099–1109. 11163185 10.1016/s0092-8674(00)00212-9

[34] Armani A, Mammi C, Marzolla V, Calanchini M, Antelmi A, et al (2010) Cellular models for understanding adipogenesis, adipose dysfunction, and obesity. Journal of Cellular Biochemistry 110: 564–572. 20512917 10.1002/jcb.22598

[35] Matsumoto T, Kano K, Kondo D, Fukuda N, Iribe Y, et al (2008) Mature adipocyte-derived dedifferentiated fat cells exhibit multilineage potential. Journal of Cellular Physiology 215: 210–222. 18064604 10.1002/jcp.21304

[36] Nobusue H, Endo T, Kano K (2008) Establishment of a preadipocyte cell line derived from mature adipocytes of GFP transgenic mice and formation of adipose tissue. Cell and Tissue Research 332: 435–446. 18386066 10.1007/s00441-008-0593-9

[37] Poloni A, Maurizi G, Leoni P, Serrani F, Mancini S, et al (2012) Human dedifferentiated adipocytes show similar properties to bone marrow-derived mesenchymal stem cells. Stem Cells 30: 965–974. 22367678 10.1002/stem.1067

[38] Song L, Tuan RS (2004) Transdifferentiation potential of human mesenchymal stem cells derived from bone marrow. FASEB journal : official publication of the Federation of American Societies for Experimental Biology 18: 980–982. 15084518 10.1096/fj.03-1100fje

[39] Webster DJ, Schneider P, Dallas SL, Muller R (2013) Studying osteocytes within their environment. Bone. 10.1016/j.bone.2013.01.004 PMC3652555 23318973

[40] Dallas SL, Bonewald LF (2010) Dynamics of the transition from osteoblast to osteocyte. Annals of the New York Academy of Sciences 1192: 437–443. 20392270 10.1111/j.1749-6632.2009.05246.x PMC2981593

[41] Veno PA, Nicolella DP, Kalajzic I, Rowe DW, Bonewald LF, et al (2007) Dynamic imaging in living calvaria reveals the motile properties of osteoblasts and osteocytes and suggests heterogeneity of osteoblasts in bone. J Bone Miner Res 22: S13.

[42] Qian H, Le Blanc K, Sigvardsson M (2012) Primary mesenchymal stem and progenitor cells from bone marrow lack expression of CD44 protein. Journal of Biological Chemistry 287: 25795–25807. 22654106 10.1074/jbc.M112.339622 PMC3406666

[43] da Silva Meirelles L, Caplan AI, Nardi NB (2008) In search of the in vivo identity of mesenchymal stem cells. Stem Cells 26: 2287–2299. 18566331 10.1634/stemcells.2007-1122

[44] Kalajzic I, Staal A, Yang WP, Wu YL, Johnson SE, et al (2005) Expression profile of osteoblast lineage at defined stages of differentiation. Journal of Biological Chemistry 280: 24618–24626. 15834136 10.1074/jbc.M413834200

[45] Bonyadi M, Waldman SD, Liu D, Aubin JE, Grynpas MD, et al (2003) Mesenchymal progenitor self-renewal deficiency leads to age-dependent osteoporosis in Sca-1/Ly-6A null mice. Proceedings of the National Academy of Sciences of the United States of America 100: 5840–5845. 12732718 10.1073/pnas.1036475100 PMC156288

[46] Park D, Spencer JA, Koh BI, Kobayashi T, Fujisaki J, et al (2012) Endogenous bone marrow MSCs are dynamic, fate-restricted participants in bone maintenance and regeneration. Cell stem cell 10: 259–272. 22385654 10.1016/j.stem.2012.02.003 PMC3652251

