# Supplementary figures and images for: Preosteocytes/Osteocytes Have the Potential to Dedifferentiate Becoming a Source of Osteoblasts
Source: PLoS One. 2013 Sep 6;8(9):e75204. doi: 10.1371/journal.pone.0075204 (PMC3765403; doi:10.1371/journal.pone.0075204)

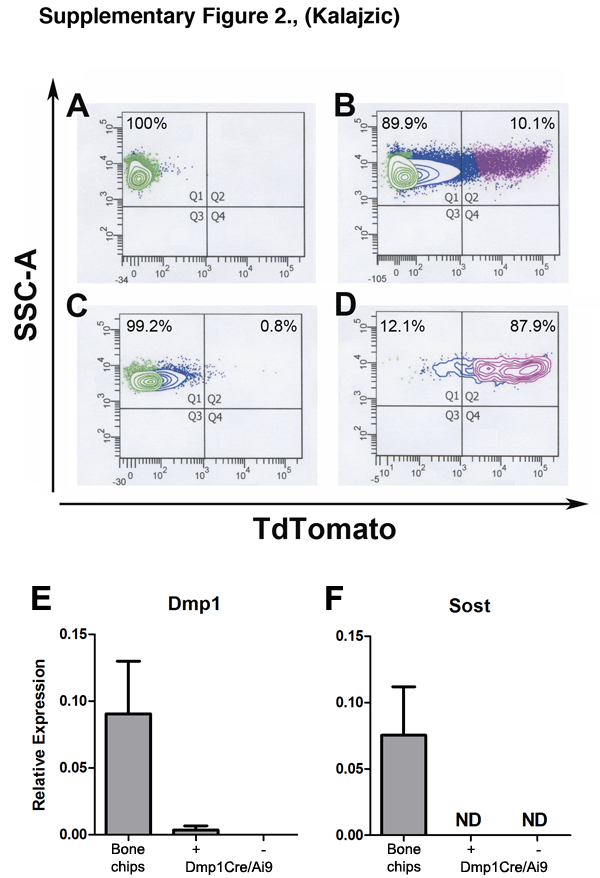

Supplement: Figure S2 — Cell sorting of BOC cultures derived from Dmp1Cre/Ai9 mice. BOC from Dmp1Cre/Ai9 mice were cultured for before FACS sorting. Nontransgenic cells were utilized as controls to preset the sorting gates (A). The proportion of Dmp1Cre/Ai9+ and Dmp1Cre/Ai9− cells is indicated (B). Reanalysis of the sorted population purity is shown for Dmp1Cre/Ai9− (C) and Dmp1Cre/Ai9+ (D) cells. Data is representative of five different sorting experiments. RNA from digested bone chips (n = 2), and from 10 day Dmp1Cre/Ai9+ and Dmp1Cre/Ai9− cultured cells immediately post sorting (n = 3) was used to assess expression of osteocyte marker genes Dmp1 (E) and Sost (F). Expression levels are presented as mean±SEM and are normalized to Gapdh expression. Changes in Dmp1 expression were not statistically significant. ND, not detectable; SSC, side scatter. (TIF) [file pone.0075204.s002.tif]

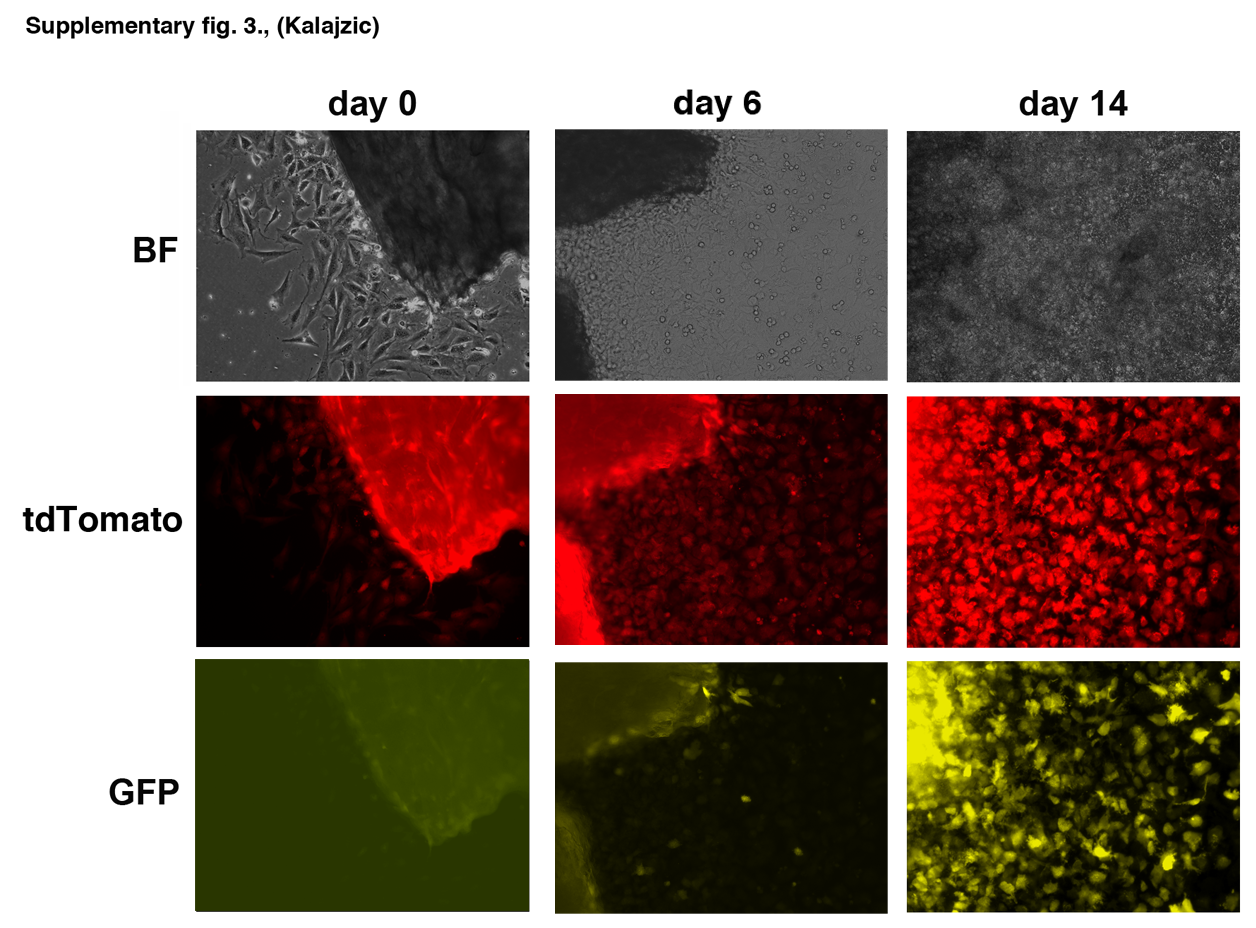

Supplement: Figure S3 — Differentiation of Dmp1Cre/Ai9+ BOC into Dmp1-GFP expressing mature osteoblast cells. Primary BOC cultures from Dmp1Cre/Ai9/Dmp1-GFP transgenic mice were cultured under basal conditions for 7 days (indicated as day 0) before treatment with osteogenic medium for 6 or 14 days. Osteogenic differentiation was confirmed by dual expression of red-labeled Dmp1Cre/Ai9 and green-labeled Dmp1-GFP cells in conjunction with the formation of mineralized nodules. BF, brightfield. (TIF) [file pone.0075204.s003.tif]
